# Pathways to Resilience: Exploring Post-Traumatic Growth in the Wake of Drug-Related Deaths

**DOI:** 10.1177/00302228241264048

**Published:** 2024-07-20

**Authors:** Ducel Jean-Berluche

**Affiliations:** 14742 University of Texas Health Science Center at San Antonio, TX, USA

**Keywords:** bereavement, post-traumatic growth, social support, grief, coping/adaptation

## Abstract

This review examines Post-Traumatic Growth (PTG) in the aftermath of Drug-Related Deaths (DRDs) amid a public health crisis underscored by an increase in overdose fatalities. It examines grief and unique challenges confronting those bereaved by DRDs, such as stigmatization, and synthesizes existing literature to elucidate pathways toward resilience and growth. Fundamental mechanisms facilitating PTG, including reframing loss, engaging in open dialogues with support, and cultivating self-compassion and hope, are highlighted, demonstrating the transformative potential of navigating bereavement with supportive communication and personal development. The review also addresses limitations within current research, such as focusing on specific bereaved populations, which may impact the generalizability of findings. Recommendations for future research include longitudinal studies and broader demographic inclusion to understand and support individuals grieving a DRD. Advocating for holistic, growth-oriented bereavement care models, this review underscores the necessity of comprehensive approaches to facilitate healing and growth in the wake of DRDs.

In 2021, an alarming surge in mortality was reported, with drug overdose deaths reaching 106,699, marking a significant public health crisis (Spencer et al., 2022). Drug-related deaths (DRDs) are classified as unnatural deaths, encompassing both sudden and unexpected fatalities (Li et al., 2003). The term encompasses fatalities resulting directly from the consumption of substances, as well as deaths among those who use substances due to violence, accidents, infectious diseases, or other health conditions potentially connected to their substance use. The sudden, unexpected loss experienced by family, friends, and community members due to these deaths has profound negative impacts, contributing to a landscape of psychological distress and interpersonal stress (Kaltman & Bonanno, 2003; Komischke-Konnerup et al., 2021; Stroebe et al., 2007). It is estimated that a death strongly impacts 10–15 people close to the deceased, implying that globally, two million people are bereaved by DRD every year (Dyregrov et al., 2020). Survivors of overdose deaths often face unique challenges, including navigating the pre-existing social dynamics related to the deceased’s substance use and confronting the stigma associated with the cause of death (Templeton et al., 2016). Research by Bottomley et al. (2022) highlights the substantial mental health burden carried by those who have lost someone to a drug overdose, indicating that this group is almost three times more likely to exhibit symptoms severe enough to meet the criteria for prolonged grief disorder (PGD), post-traumatic stress disorder (PTSD), and major depressive disorder (MDD) compared to those grieving sudden natural losses.

Adaptation following the loss of a loved one is inherently personal, shaped by many factors, including individual characteristics, the support systems in place, and cultural norms (Jordan & Litz, 2014). Sudden loss disrupts the bereaved ability to find meaning in their loss, where failure to make sense of the event can intensify distress, lead to maladaptive cognitions, and exacerbate peritraumatic distress (Boelen, 2015). Despite the complexities of the grieving process, which can be influenced by factors such as the relationship of the bereaved to the deceased, their age, gender, available social support, spirituality, and cultural guidelines, there remains a potential for resilience and growth (Kaltman & Bonanno, 2003; Tedeschi & Calhoun, 2004).

Post-traumatic growth (PTG) refers to the positive psychological changes that occur as individuals navigate the challenges posed by life-altering crises (Tedeschi & Calhoun, 2004). This phenomenon, characterized by significant shifts in life appreciation, personal relationships, sense of self, recognition of new possibilities, and spiritual development, arises not directly in response to the trauma but through grappling with its aftermath (Calhoun et al., 2010). It is important to note that experiencing traumatic events does not guarantee PTG; the emergence of growth is contingent upon the individual’s engagement in reflective processes that reconstruct personal beliefs, goals, and life narratives post-trauma.

The concept of PTG, especially in the context of bereavement following a DRD, remains underexplored (Titlestad et al., 2021). The limited research into bereavement due to DRDs suggests an insufficiency to support this population effectively. The only existing systematic review on DRD points to an emotional and existential overload in the bereaved but lacks understanding and help from the support systems (Titlestad et al., 2021). There is a critical need to deepen our understanding of the bereavement-related needs of survivors of sudden loss to inform both future research and the development of support mechanisms by healthcare providers and policymakers. Therefore, this review aims to explore and synthesize existing literature to better understand PTG in this population better contribute insights that may aid in addressing this ongoing public health crisis.

## Overview of Drug-Related Deaths

The United States has witnessed a significant surge in fatal overdoses over the last two decades, markedly contributing to the premature loss of life (Case & Deaton, 2015; Dowell et al., 2017; Jalal et al., 2018). Often, the magnitude of drug overdose deaths is highlighted by comparing them to other causes of mortality, illustrating their effect on public health. For instance, it is frequently noted that opioid overdoses now result in more annual deaths than those caused by motor vehicle accidents (Rudd et al., 2016). The escalation in opioid-related deaths has occurred in three distinct phases, beginning with prescription opioids, then transitioning to heroin, and most recently involving synthetic opioids like fentanyl (Ciccarone, 2019). Additionally, there has been a noticeable increase in fatalities attributed to other substances, including prescription medications such as benzodiazepines and stimulants, alongside illicit drugs like cocaine and methamphetamine (Bachhuber et al., 2016; Jones et al., 2015; Scholl et al., 2018).

## Impact of Drug-Related Deaths on Survivors

### Psychological and Emotional Toll

The psychological and emotional impact of DRDs on survivors is multifaceted, as evidenced by an array of studies exploring the bereavement experiences of affected families. Christiansen et al. (2020) found that parents who lost a child to a DRD faced a higher mortality rate from natural causes and notably high external cause mortality within the first two years after the loss compared to non-bereaved parents and those bereaved by other causes. This heightened vulnerability underscores the acute stress and health risks associated with such a bereavement. Compounding the issue, research indicates that a significant proportion of bereaved individuals—ranging from 30 to 70% following sudden and violent losses—develop Prolonged Grief Disorder (PGD), a rate much higher than the 10–15% typically observed after expected losses (Djelantik et al., 2020; Lundorff et al., 2017). Specifically, Titlestad and Dyregrov (2022) observed that 26% of family members bereaved by DRDs exhibited high levels of prolonged grief symptoms, with parents mainly affected.

A systematic review by Titlestad et al. (2021) identified critical themes in the bereavement experiences of DRD family members, including an emotional roller coaster, a pervasive lack of understanding of the social world, and challenges in meaning-making. These themes highlight the emotional and existential burden borne by survivors, compounded by stigmatization and a dearth of empathetic support. Similarly, da Silva et al. (2007) revealed that families unaware of their loved one’s substance use faced shock and a complex mix of anger, guilt, and helplessness upon their loss. In contrast, families who knew about the drug use experienced ambivalent feelings of grief mixed with relief, indicative of a “veiled preparation” for the potential loss. In a related study, Schlosser and Hoffer (2022) provided insight into how people who use opioids (PWUO) navigate the bereavement of overdose deaths within their community. Their interviews illustrated overdose death as a constant threat in the lives of PWUO, influencing their drug use behaviors—some increased their use. In contrast, others adopted safer practices out of fear and loss. This pervasive fear of death and its impact on drug use behaviors underscores the complex interplay between grief and survival strategies among those directly affected by the opioid crisis.

## Stigma and Social Dynamics Surrounding Drug-Related Deaths

### Stigmatization

The stigma surrounding DRDs significantly influences the bereavement process, compounding the grief of those left behind. Societal perceptions can vary significantly with the cause of death, with deaths by suicide and drugs considered low status, thereby exacerbating the challenges bereaved individuals face during their grieving process. This societal ranking underscores the additional burden placed on those mourning DRDs as they navigate not only their loss but also the diminished societal sympathy and support. Kheibari et al. (2021) further highlight the negative stigmatization associated explicitly with death by opioid overdose. Their research underscores the pervasive societal stigma that not only impacts the deceased’s memory but also significantly affects the mourning process of their loved ones, isolating them and complicating their grief.

In exploring the experiences of parents bereaved by drug deaths, Titlestad et al. (2020) identified stigmatization as a central theme among participants. Parents reported facing both societal stigma and self-stigma, with societal attitudes often reflecting the belief that substance use disorder and its fatal consequences are self-inflicted. This stigma manifests in various ways, affecting the bereaved interactions with their social circles and the support they receive, or lack thereof. In a related study, Dyregrov and Selseng (2021) examined the breadth of stigmatization experienced by individuals grieving a DRD, finding that it emanated not just from personal acquaintances but also from professionals and society at large. The stigma was communicated both directly and indirectly, with bereaved individuals encountering dehumanizing labels, implicit stigma, and attributions of blame towards the deceased. Such stigmatization not only alienates the bereaved but also suggests a societal view that death is an inevitable or even preferable outcome, further complicating the grieving process.

Feigelman et al. (2011) provide comparative insight into the grieving experiences of parents who have lost their children to various causes, including DRDs. Their findings reveal that parents mourning a DRD face significantly more grief and mental health challenges, partly due to societal stigmatization against drug use. This public stigmatization leads to less compassionate responses and support for the bereaved, highlighting the critical need for societal change to address the unique challenges faced by this population.

### Challenges in Accessing Support

Navigating the aftermath of a substance-related death presents significant challenges for the bereaved, particularly when seeking support from services designed to assist them during their time of loss. Valentine et al. (2018) conducted qualitative interviews with 106 adults bereaved by substance use, revealing that many encountered insensitive, judgmental, and abrupt responses from practitioners. These negative experiences seem to stem from the broader stigma associated with substance use, extending its reach into the very services that should offer solace and support. Such interactions not only fail to meet the emotional and practical needs of the bereaved but also risk reinforcing their feelings of isolation and marginalization. Conversely, Walter et al. (2017) explored the experiences of loss and support among adults bereaved through substance use and found that small acts of kindness from those encountered in the aftermath of death could significantly mitigate the effects of stigma. These gestures—simple yet profound—could influence whether bereaved individuals felt they mattered and were deserving of empathy and support. This study underscores the potential for positive change in how services engage with and support those bereaved by substance use, highlighting the importance of compassion and understanding in overcoming the barriers posed by societal stigma.

## Method

A literature search strategy was conducted across databases to enhance the comprehensiveness and precision of the investigation into PTG following DRDs. The databases included PubMed, PsycINFO, CINAHL, and Scopus. The search utilized a combination of keywords and phrases designed to capture the essence of the research interest. These keywords included “post-traumatic growth,” “drug-related deaths,” “bereavement,” “resilience,” and “grief,” among others, used in various combinations to ensure a comprehensive retrieval of relevant literature.

The inclusion criteria were strictly defined to ensure the relevance and quality of the selected studies. The review focused exclusively on peer-reviewed articles published in English. The search scope was narrowed to empirical studies directly exploring bereavement resulting from DRDs, ensuring that the findings were grounded in observed and analyzed data relevant to the study area. This approach allowed the opportunity to concentrate on research that provided insights into the psychological aftermath and growth experiences of individuals facing the specific trauma of losing someone to drug-related circumstances.

The exclusion criteria were designed to filter out studies that did not align with the research objectives. Non-empirical studies, including theoretical papers and opinion pieces, were excluded to maintain a focus on original research findings. Articles not published in English were also excluded to ensure that the review process was feasible and that findings were accessible to an English-speaking audience. Additionally, studies that addressed bereavement but were unrelated to DRDs were omitted, as the interest was specifically in understanding the unique experiences of growth and adaptation following the loss attributed to drug use.

The search strategy was executed precisely, and the results were screened at two levels. Initially, titles and abstracts were reviewed to assess their relevance based on the predefined inclusion and exclusion criteria. Subsequently, full texts of potentially relevant articles were thoroughly examined to confirm their suitability for inclusion in the review. This two-step screening process ensured that only studies directly contributing to the understanding of PTG in the context of DRDs were selected for analysis.

## Post-traumatic Growth following Drug-Related Deaths

Exploring the pathways toward resilience and growth in the aftermath of DRDs reveals both the challenges and potential for transformation that bereaved individuals may experience. Research in this area, focusing on PTG, provides valuable insights into how survivors may navigate their grief and find new meaning in life after such a tragic loss. In a study by O’Callaghan et al. (2022), they explored the experience of PTG among families who have lost a member to a DRD. Their study identified vital mechanisms facilitating growth, including reframing loss, fostering open dialogues with social support, and discovering new purposes in life. These strategies emphasize the importance of adaptive narrative reshaping and supportive communication in navigating the complexities of grief and facilitating PTG.

In a related study by Hill and O’Brien (2023), these researchers investigated grief outcomes among 159 individuals mourning an opioid-related death, focusing on the predictive roles of disenfranchised grief, social support, and coping strategies. The findings revealed that when predicting prolonged grief, avoidant emotional coping (β = 0.55) alone accounted for unique variance. Active emotional coping (β = 0.28) and problem-focused coping (β = 0.40) explained unique variance in PTG. The differentiation in this study demonstrates the effectiveness of proactive engagement with grief as a catalyst for growth. Moreover, Feigelman et al. (2018) conducted 11 in-depth qualitative interviews with bereaved parents, who reported anxiety-inducing interactions with police or medical personnel, difficulties sharing death cause information with socially significant others, and longer-term problems from routine interactions. The challenges described eventually led to a turning point in the development of PTG. Openly disclosing the nature of their child’s death seemed to have been an essential building block for their healing.

Sperandio et al. (2021) conducted a study that examined the interaction between hope and self-compassion and their collective impact on PTG among those grieving a DRD. The study offered critical insight into the psychological constructs that underpin the journey toward recovery and growth in the aftermath of such profound losses. Significantly, the research demonstrated that self-compassion is not merely a beneficial trait but a predictor for the emergence of PTG, operating independently from hope. The findings underscore the transformative power of self-compassion in the bereavement process, suggesting that individuals who practice kindness and understanding towards themselves in times of suffering are more likely to navigate their grief toward pathways of growth. Furthermore, hope emerged as a critical mediator in this complex interplay, enhancing the relationship between self-compassion and PTG. This implies that while self-compassion lays the foundation for a constructive internal dialogue in the face of loss, hope acts as the catalyst that propels the bereaved towards envisioning a future where healing and growth are possible. The mediating role of hope illuminates its importance not only as a forward-looking belief in positive outcomes but also as an essential element that amplifies the effects of self-compassion on PTG. The study by Sperandio et al. (2021) enriches our understanding of the factors contributing to PTG in the context of DRD bereavement. It highlights the therapeutic value of cultivating self-compassion and hope as vital components of the healing journey.

## Discussion

The exploration of PTG following DRDs reveals critical insights into the resilience and transformative potential within individuals navigating the aftermath of tragic losses. The findings in this review highlight mechanisms such as reframing loss, engaging in open dialogues with supportive communication, and fostering self-compassion and hope as facilitators of PTG, which is consistent with previous studies (Büchi et al., 2007, 2009; Zhou et al., 2018). Reframing loss highlights the power of narrative in the healing process, suggesting that altering one’s perception of the loss can significantly influence the bereavement trajectory. This can be seen in previous research demonstrating a positive relationship between meaning reconstruction and PTG among those grieving a loss (Bogensperger & Lueger-Schuster, 2014). On the other hand, engaging in open dialogues with supportive communication fosters a sense of community and shared understanding, offering the bereaved a platform to express their grief and vulnerabilities, thereby mitigating the sense of isolation often associated with such losses. This is consistent with prior research demonstrating how a supportive environment can facilitate PTG (Gerrish et al., 2014). Furthermore, the cultivation of self-compassion and hope emerges as fundamental in navigating the complexities of grief, with hope acting as a crucial mediator that amplifies the positive impact of self-compassion on PTG. This intricate interplay between self-compassion and hope suggests a dynamic process wherein the bereaved learn to extend kindness to themselves, fostering a hopeful outlook toward the future and facilitating a constructive engagement with their grief. Previous research has shown how self-compassion can be critical in facilitating PTG (Munroe et al., 2022).

In addressing the grief following Drug-Related Deaths (DRDs), there is a compelling need for bereavement support services to integrate and actively promote adaptive mechanisms such as self-compassion, hope, and narrative reframing, shifting toward a holistic, growth-oriented model of care. Incorporating grief-focused Cognitive Behavioral Therapy (CBT), as demonstrated by Komischke-Konnerup et al. (2023), offers a structured approach to cognitive restructuring, allowing individuals to reframe their loss and reconceptualize self-identities, aligning with the adaptive mechanisms essential for PTG. Additionally, engaging bereaved individuals in peer support services, as evidenced by Bartone et al. (2019), provides a unique platform for sharing experiences and receiving empathetic support, which has been shown to reduce grief symptoms and enhance well-being. The collaborative application of grief-focused CBT alongside peer support summarizes a multidisciplinary strategy that addresses the psychological, social, and potentially spiritual facets of grieving. It fosters an environment where the bereaved can navigate their grief with hope and compassion, setting a foundation for lasting resilience and transformative growth.

While the studies reviewed provide valuable insights into the potential for PTG following DRDs, several limitations must be acknowledged. Firstly, the variability in the definition and measurement of PTG across studies may impact the generalizability of the findings. Additionally, relying on self-reported data could introduce bias, as individuals may have differing interpretations of what constitutes growth. Furthermore, most of the research focuses on specific populations, such as parents or family members, which may not fully represent the diverse experiences of all those bereaved by DRDs. This indicates a need for broader research encompassing various demographic and relational contexts.

Given the identified limitations, there are several recommendations for future research. There is a need for longitudinal studies that follow bereaved individuals over time to understand better the trajectory of PTG and the lasting impact of bereavement interventions. Research should also incorporate more standardized measurements of PTG to enhance comparability across studies. Expanding the focus to include a broader range of bereaved individuals beyond immediate family members could provide a more comprehensive understanding of the PTG process in the context of DRDs. Moreover, investigating the role of cultural, social, and environmental factors in shaping the experience of PTG could offer deeper insights into how best to support bereaved individuals across diverse contexts.

The journey through grief following a DRD presents both significant challenges and opportunities for personal growth. The current body of research on PTG in the context of DRDs highlights key factors that facilitate this growth, including the critical roles of reframing loss, fostering open communication with support, and nurturing self-compassion and hope. Despite the limitations of existing studies, the potential for PTG offers a hopeful perspective for those navigating the complexities of bereavement. Future research should aim to build upon these findings, seeking to understand the nuanced processes that contribute to resilience and growth to enhance support for those bereaved by DRDs.

## Data Availability

Data sharing is not applicable to this article as no new data were created or analyzed in this study.
